# Histamine Recycling Is Mediated by CarT, a Carcinine Transporter in *Drosophila* Photoreceptors

**DOI:** 10.1371/journal.pgen.1005764

**Published:** 2015-12-29

**Authors:** Ying Xu, Futing An, Jolanta A. Borycz, Janusz Borycz, Ian A. Meinertzhagen, Tao Wang

**Affiliations:** 1 School of Life Sciences, Beijing Normal University, Beijing, China; 2 National Institute of Biological Sciences, Beijing, China; 3 School of Life Sciences, Beijing University, Beijing, China; 4 Department of Psychology and Neuroscience, Life Sciences Centre, Dalhousie University, Halifax, Nova Scotia, Canada; New York University, UNITED STATES

## Abstract

Histamine is an important chemical messenger that regulates multiple physiological processes in both vertebrate and invertebrate animals. Even so, how glial cells and neurons recycle histamine remains to be elucidated. *Drosophila* photoreceptor neurons use histamine as a neurotransmitter, and the released histamine is recycled through neighboring glia, where it is conjugated to β-alanine to form carcinine. However, how carcinine is then returned to the photoreceptor remains unclear. In an mRNA-seq screen for photoreceptor cell-enriched transporters, we identified CG9317, an SLC22 transporter family protein, and named it CarT (Carcinine Transporter). S2 cells that express CarT are able to take up carcinine *in vitro*. In the compound eye, CarT is exclusively localized to photoreceptor terminals. Null mutations of *cart* alter the content of histamine and its metabolites. Moreover, null *cart* mutants are defective in photoreceptor synaptic transmission and lack phototaxis. These findings reveal that CarT is required for histamine recycling at histaminergic photoreceptors and provide evidence for a CarT-dependent neurotransmitter trafficking pathway between glial cells and photoreceptor terminals.

## Author Summary

Neurotransmitter transporters that remove neurotransmitters and recycle them after their release have particular importance at visual synapses, which must signal at high frequencies and therefore required rapid clearance of neurotransmitters from the synaptic cleft. In this study, we identified a SLC22 family transporter, CarT, in the visual system of *Drosophila*, which is exclusively located to photoreceptor terminals in the lamina neuropil and is responsible for taking up carcinine, an inactive histamine metabolite, from surrounding glia. Loss of CarT disrupts the regeneration of histamine and blocks neurotransmission at photoreceptor cell synapses. Our work provides direct evidence for a local histamine recycling pathway between glial cells and photoreceptor terminals, and shows that a CarT-dependent histamine/carcinine shuttle pathway is critical for maintaining the normal histamine content of neurons.

## Introduction

Histamine is an important chemical messenger known to be involved in a broad spectrum of biological processes such as inflammation and gastric acid secretion. It is also recognized as an important neurotransmitter [1]. Recycling histamine at histaminergic synapses is a key event both in maintaining synaptic transmission and in terminating histamine’s action on postsynaptic neurons. The *Drosophila* visual system uses histamine as the neurotransmitter at photoreceptor synapses, and provides a good genetic model for studying histamine, its metabolism and recycling [2]. The compound eye of *Drosophila* is composed of ~800 ommatidia, each of which contains eight photoreceptor cells. Of the latter, R1-R6 photoreceptors in each ommatidium project axons from the retina to the underlying lamina neuropil, where they are organized into synaptic modules called cartridges. R7/R8 photoreceptors project axons to the second neuropil, the medulla [3–6]. In lamina cartridges, three epithelial glial cells normally envelop six photoreceptor terminals [7].

Although the synthesis of histamine from histidine occurs *de novo* under the action of histidine decarboxylase (Hdc) in photoreceptor cells, recycling of histamine is reported to be the dominant pathway for maintaining the histamine content in photoreceptors [8,9]. Both pathways, *de novo* synthesis and recycling, are required to maintain an adequate content of histamine in photoreceptor cells. Disrupting either pathway affects visual synaptic transmission in *Drosophila* in the long term [8,10]. Upon light stimulation, photoreceptor terminals release histamine as a neurotransmitter, which activates histamine-gated chloride channels (HisClA) on large monopolar cells (LMCs) in the lamina and hyperpolarizes these postsynaptic neurons [2,4,11]. After its release, histamine is taken up by lamina glia and conjugated to β-alanine, converting it to carcinine by the N-β-alanyl-dopamine synthase, Ebony, which is expressed in epithelial glia [10,12,13]. The metabolized histamine conjugate, carcinine, is then transported back into the photoreceptors and hydrolyzed back to histamine by Tan, an N-β-alanyl-dopamine hydrolase[10,14]. Despite knowledge of these pathways, little is known about the critical step by which carcinine is transported back to the photoreceptors. It has been proposed that the gene *inebriated* (*ine*) might encode a carcinine neurotransmitter transporter in photoreceptor cells to take up carcinine from synaptic cleft [15]. However, in this study, we show that Ine fails to function in any clear way in photoreceptor cells. In addition, the cellular location for carcinine uptake, the trafficking route by which it is returned to the photoreceptor cells where the Tan enzyme has to act, and the transporters responsible for carcinine uptake, all remain controversial. Recently, it has been suggested that metabolites of histamine are transported between glia and the cell bodies of photoreceptors through networks of intercellular gap junctions [9].

We identified a photoreceptor cell-enriched neurotransmitter transporter, CarT, which is able to transport carcinine across the membranes of photoreceptors. CarT is predominantly localized to photoreceptor terminals. The *cart* mutant flies are defective in photoreceptor synaptic transmission, and as a result lack phototaxis. In addition, we found that a human homologue of CarT, Organic Cation Transporter (OCT2), can also transport carcinine *in vitro* and is thus able to reverse synaptic transmission defects in *cart* mutant flies. We therefore propose the presence of a novel pathway for histamine recycling, in which the carcinine transporter CarT efficiently takes up carcinine that is released locally from glial cells lying in close vicinity to photoreceptor terminals.

## Results

### *CG9317* encodes a photoreceptor cell-enriched transporter

**Fig 1:**
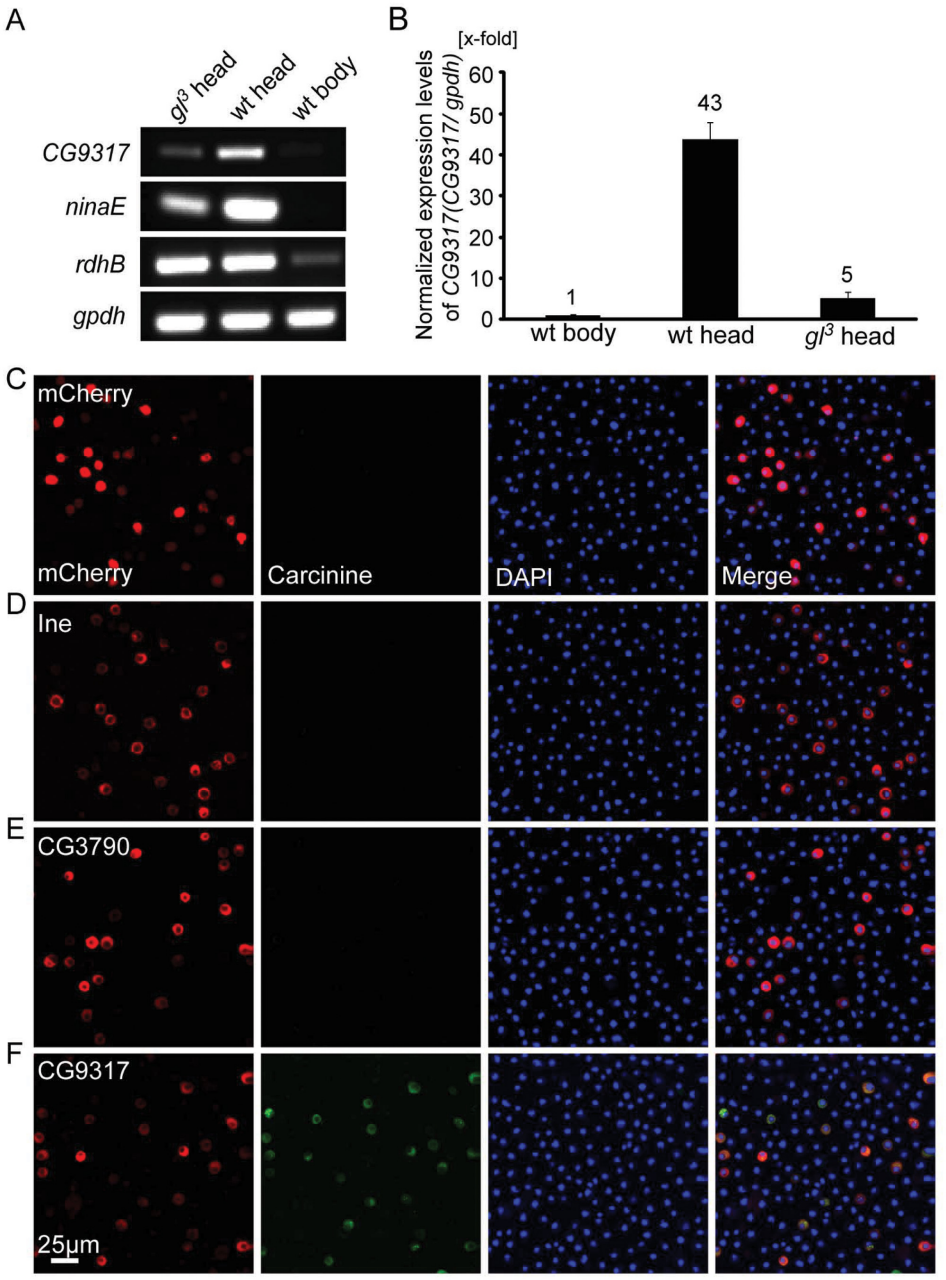
CG9317 is photoreceptor cell-enriched carcinine transporter. (A-B) Photoreceptor cells express CG9317 at a high level. (A) qPCR experiments show that *CG9317* expression is enriched in wild-type (wt: *w* ^*1118*^) heads compared with *gl* ^*3*^ heads or wild-type bodies. (B) The ratio of *CG9317* transcript levels versus *gpdh* transcript levels was determined using quantitative PCR. The mRNA level was normalized to the wild-type body, relative to which the *CG9317* transcript levels were increased about 43 fold and 5 fold in the heads of wild-type and *gl* ^*3*^ mutant flies respectively. Error bars indicate the SD. (C-F) S2 cells transiently expressed (C) mCherry, (D) Ine-mCherry, (E) CG3790-mCherry or (F) CG9317-mCherry. Carcinine was added to the culture medium at a final concentration of 20μm. Cells were labeled with rabbit anti-carcinine (green) and DAPI (blue). The mCherry (red) signal was observed directly. Scale bar, 25μm.

Given that the histamine/carcinine shuttle in the visual system occurs between photoreceptors and surrounding glia cells [7], and that the enzyme Tan responsible for hydrolyzing carcinine to release histamine is exclusively expressed in photoreceptor cells, we assumed that the neurotransmitter transporter responsible for taking up carcinine must be enriched in photoreceptor cells. The gene *glass* (*gl*) gene encodes a zinc finger transcription factor, and *glass* mutations specifically remove photoreceptor cells, but leave other cell types intact. Mutations of *glass* specifically remove photoreceptor cells, and thus largely abolish the expression of mRNA transcripts of photoreceptor-enriched genes, such as the gene encoding major rhodopsin *neither inactivation nor afterpotential E* (*ninaE*). Expression of *ninaE* is greatly reduced in the heads of *gl* ^*3*^ flies relative to wild-type (*w* ^*1118*^) heads (Fig 1A).

By comparing mRNAs isolated from wild-type heads with *gl* ^*3*^ heads or wild-type bodies, we identified a list of genes that are expressed predominantly in photoreceptor cells. We examined both this RNA-seq data and a DNA microarray data set, which screened for genes expressed predominantly in photoreceptor cells and the compound eyes respectively [16]. This enabled us identify candidate genes that might encode the carcinine transporters. *CG9317* and *CG3790* are both candidate genes for eye-enriched neurotransmitter transporters. Both proteins share significant amino acid identities with the mammalian solute carrier family 22 (SLC22) family proteins, including the mouse OCT2 and OCT3 (S1 Fig). The expression of *CG9317* mRNA was greatly reduced in *gl* ^*3*^ fly heads, indicating that *CG9317* is expressed predominantly in photoreceptor cells (Fig 1A and 1B). In contrast, the expression levels of the retinal pigment cell marker gene *retinol dehydrogenase B* (*rdhB*) remain unchanged for both *gl* ^*3*^ flies and wild-type flies (Fig 1A) [17].

### CG9317 is a carcinine transporter *in vitro*

We next conducted *in vitro* experiments to examine whether CG9317 and CG3790 can transport carcinine. We expressed mCherry-tagged proteins in S2 cells, and used immunolabeling to examine the intracellular signals for histamine or carcinine. Carcinine or histamine was added to the medium to yield final concentrations of 20μM. After three-hour incubations, the intracellular carcinine or histamine signal was examined. No transporter activity for either carcinine or histamine was detected in S2 cells expressing mCherry alone (Fig 1C and S2A Fig). There was no immunosignal for either carcinine or histamine after expressing Ine, which indicates the probability that Ine does not transport either carcinine or histamine under the conditions tested (Fig 1D and S2B Fig). We next examined the candidate carcinine transporters that are highly expressed in eyes, including CG9317 and CG3790 [16]. CG3790 failed to transport either carcinine or histamine (Fig 1E and S2C Fig). We confirmed these results by using a specific rat anti-carcinine antibody from a different source [18] (S3 Fig). In contrast, a clear immunosignal for carcinine but not histamine was detected in cells expressing CG9317 (Fig 1F and S2D Fig). When we expressed histidine decarboxylase (Hdc) in S2 cells, immunosignal for histamine was observed, which served as a positive control, validating our *in vitro* histamine immunolabeling method (S2E Fig). These findings suggest that *CG9317* encodes a carcinine transporter, which we therefore named CarT (Carcinine Transporter).

### Visual synaptic transmission is defective in *cart* mutants

**Fig 2:**
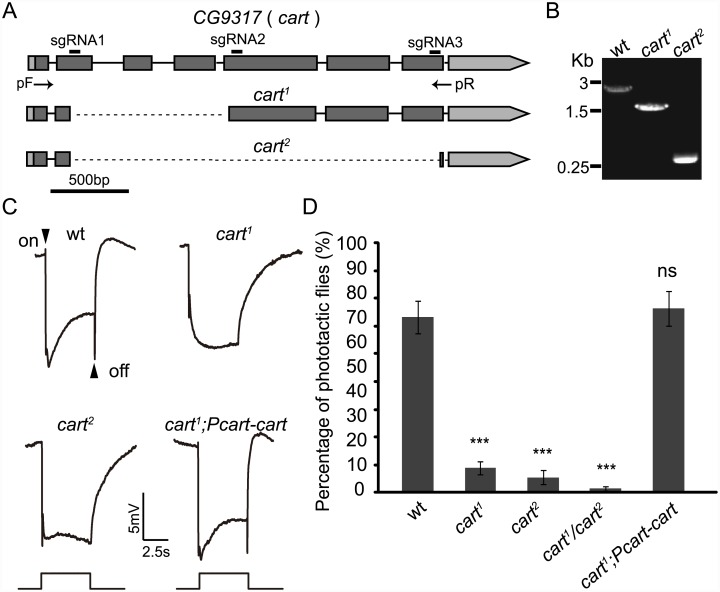
Mutations in *cart* eliminate “on” and “off” transients in ERG recordings. (A) Schemes for *cart* deletion by sgRNA targeting. The organization of the *cart* locus and the expected structure of the deletion alleles (*cart* ^*1*^ and *cart* ^*2*^) are shown. Boxes represent exons, and deep gray boxes represent the coding region. The sgRNA1 and sgRNA2 primer pair was used to generate the *cart* ^*1*^ mutation. The sgRNA1 and sgRNA3 primer pair was used to generate the *cart* ^*2*^ mutation. The positions of the DNA primers used for PCR (arrows) are indicated. (B) PCR products obtained from successful *cart* deletion mutants. The agarose gel electrophoresis of PCR products using the primers indicated in (A) (pF and pR) is shown and genomic DNA templates prepared from wt (*w* ^*1118*^), *cart* ^*1*^ and *cart* ^*2*^ flies. (C) ERG recordings from wt (*w* ^*1118*^), *cart* ^*1*^, *cart* ^*2*^ and *cart* ^*1*^; *Pcart-cart* flies. Flies (~1 d after eclosion) were dark adapted for 1 min and subsequently exposed to a 5s pulse of orange light. (D) Phototaxis assays revealed a difference in behavior between wt and *cart* mutant flies. Error bars: SD; significant differences between mutant and wt flies were determined using unpaired *t*-tests (*p < 0.05; **p < 0.01; ***p < 0.001; ns, not significant).

To characterize the requirement for CarT in transmitting visual signals, we generated two different null mutations in the *cart* gene using the CRISPR-associated single-guide RNA system (Cas9)(Fig 2A)[19]. We identified fly lines containing these *cart* ^*1*^ and *cart* ^*2*^ mutations by PCR using genomic primers outside of the deleted regions (Fig 2A). Full-length PCR products were detected in wild-type flies, whereas shorter PCR products were detected in the *cart* ^*1*^ and *cart* ^*2*^ mutant lines, indicating the disruption of the *cart* locus in *cart* ^*1*^ and *cart* ^*2*^ flies (Fig 2B). The *cart* genomic region in both mutations was sequenced, and 1112 and 2344 bp fragments were deleted in *cart* ^*1*^ and *cart* ^*2*^ mutants respectively (S4 Fig).

As *cart* mutants were not lethal, so we undertook electroretinogram (ERG) recordings directly. ERG recordings are extracellular recordings that measure the summed responses of all retinal cells in response to light. Upon exposure to light, an ERG recording from a wild-type fly contains a sustained depolarizing response from the photoreceptors, and “on” and “off” transients originating from synaptic transmission to the lamina [20] (Fig 2C). Mutations with defective synaptic transmission have obvious reductions in their “on” and “off” transients [6]. As in mutants of genes involved in histamine recycling, ERG transients were not observed in *cart* ^*1*^, *cart* ^*2*^, or *cart* ^*1*^/ *cart* ^*2*^ mutant flies (Fig 2C). Phototaxis is a visual behavior that requires the integrity of the neuron circuits of the visual system [21], and defective synaptic transmission of visual signals results in poor phototaxis [22]. Significantly reduced phototaxis was associated with all the *cart* ^*1*^, *cart* ^*2*^, and *cart* ^*1*^/*cart* ^*2*^ mutations (Fig 2D). To further confirm that the loss of visual synaptic transmission resulted from mutations of the *cart* locus, we generated a *Pcart-cart* transgenic fly line expressing the *cart* cDNA under the control of the *cart* promoter. The *Pcart-cart* transgene reversed the loss of “on” and “off” transients and restore phototaxis in the *cart* ^*1*^ mutant flies (Fig 2C and 2D).

### CarT functions in photoreceptor cells

Tan, the hydrolase that deconjugates carcinine and releases histamine, localizes to photoreceptor cells and functions downstream of the transport of carcinine. A carcinine transporter coupled with Tan’s action should therefore be expressed and should function in photoreceptor cells. It has been suggested that *ine* encodes a putative carcinine neurotransmitter transporter in photoreceptor cells [15]. We used the *eyeless-GAL4 UAS-FLP* (EGUF)*/hid* technique to generate genetically mosaic flies [23]. The compound eyes of these mosaic flies comprise cells homozygous for a selected mutation, but forming part of an entire mosaic fly that is elsewhere heterozygous for the mutation. Therefore, if Ine functions in the compound eye of *Drosophila*, eye-specific mutations of *ine* in *ine* mosaic flies should mirror at least the same ocular defects in synaptic transmission as those present in the *ine* mutants.

**Fig 3:**
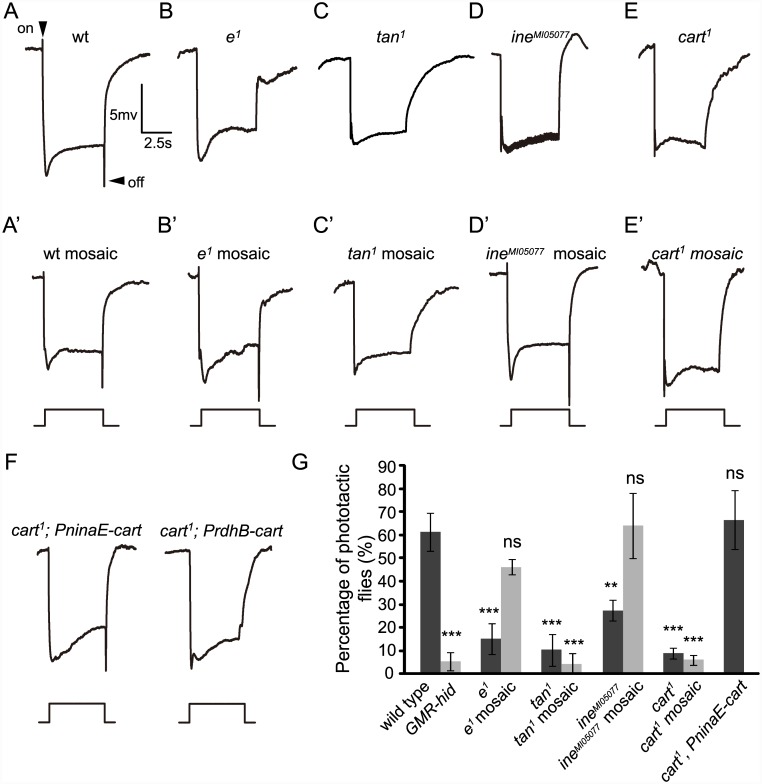
CarT is required for synaptic transmission in photoreceptor cells. (A-E) ERG paradigm that elicits “on” and “off” transients (arrows) in (A) wt (*w* ^*1118*^) flies but not in (B) *e* ^*1*^, (C) *tan* ^*1*^, (D) *ine* ^*MI05077*^ or (E) *cart* ^*1*^ mutant flies. Flies (~1 d after eclosion) were dark adapted for 1 min and subsequently exposed to a 5s pulse of orange light. (A’-E’) “Off” transients were observed in the wt mosaic, *e* ^*1*^ and *ine* ^*MI05077*^ mosaic eyes but not in *tan* ^*1*^ or *cart* ^*1*^ mosaic eyes. The genotypes are as follows: (A’: wt mosaic) *ey-flp; FRT40A/GMR-hid CL FRT40A*. (B’: *e* ^*1*^ mosaic) *ey-flp; FRT82B e* ^*1*^ */FRT82B GMR-hid CL*. (C’: *tan* ^*1*^ mosaic) *tan* ^*1*^ *FRT19A/GMR-hid FRT19A; ey-flp*. (D’: *ine* ^*MI05077*^ mosaic) *ey-flp; ine* ^*MI05077*^ *FRT40A /GMR-hid CL FRT40A*. (E’: *cart* ^*1*^ mosaic mosaic) *ey-flp; cart* ^*1*^ *FRT40A /GMR-hid CL FRT40A*. (F) Expression of CarT in photoreceptor cells (*cart* ^*1*^ *; PninaE-cart*), but not in pigment cells (*cart* ^*1*^ *; PrdhB-cart*), restored the “on” and “off” transients in *cart* ^*1*^ flies. (G) Quantification of phototaxis of flies with the indicated genotypes. Error bars represent SD. Significant differences between mutant and wt flies were determined using unpaired *t*-tests (*p < 0.05; **p < 0.01; ***p < 0.001; ns, not significant).

We observed that ERG recordings from wild-type eyes have normal “on” and “off” transients (Fig 3A and 3A’). Mutations in both the *ebony* (*e* ^*1*^) and the *tan* (*tan* ^*1*^) genes disrupt histamine recycling and this results in the loss of “on” and “off” transients in their ERG recordings (Fig 3B and 3C) [10]. As expected, *e* ^*1*^ mosaic flies in which all photoreceptors were homozygous mutant for *ebony* had wild-type “on” and “off” transients. This is because Ebony is not required in the photoreceptors but is required in glial cells lying outside them (Fig 3B’). As Tan functions in the photoreceptor cells of the compound eye, the *tan* ^*1*^ mosaic which lacks *tan* expression in the photoreceptors displayed reduced “on” and “off” transients (Fig 3C’).

The ERG responses of *ine* mutants (*ine* ^*MI05077*^) contain prominent oscillations superimposed on the sustained depolarizating response and they also have reduced “on” and “off” transients (Fig 3D) [15]. The latter phenotype indicates impaired photoreceptor synaptic transmission. However, as with the *ebony* mutants, heterozygous flies with homozygous *ine* mutant compound eyes (*ine* mosaic flies) had wild-type ERG responses with normal “on” and “off” transients (Fig 3D’), indicating that Ine does not function obligatorily in photoreceptor cells. Therefore, it is unlikely that Ine is directly or necessarily responsible for carcinine uptake at the photoreceptor cell membrane, as previously suggested. Its possible role as a transporter elsewhere is not addressed by these experiments.

Given that expression of the *cart* gene is enriched in photoreceptor cells, we assumed that CarT is required in photoreceptor cells for synaptic transmission. As expected, homozygous *cart* ^*1*^ mutant eyes lacked “on” and “off” transients despite the heterozygous background elsewhere (Fig 3E and 3E’). This finding indicates that CarT functions in the compound eyes. Photoreceptor cells and retinal pigment cells are the two major cell types in the compound eye. To confirm the retinal cell type in which CarT functions, we expressed CarT specifically in photoreceptor cells using the *ninaE* promoter or in retinal pigment cells using the *rdhB* promoter [17] [24]. Photoreceptor-enriched expression of CarT by *PninaE-cart* restored both the “on” and “off” transients and phototaxis in *cart* ^*1*^ mutant flies, whereas expression of CarT in pigment cells through *PrdhB-cart* did not (Fig 3F and 3G). These results strongly support the interpretation that CarT functions in photoreceptor cells to maintain synaptic transmission.

In addition, we extended these ERG results by phototaxis assays. Wild-type flies displayed positive phototactic behavior, whereas flies that were homozygous mutant for *ebony*, *tan*, *ine*, or *cart* all displayed poor phototaxis, indicating that these genes are required for visual synaptic transmission (Fig 3G). Consistent with the ERG results, phototaxis was significantly reduced in the mosaic eyes of *tan* ^*1*^ and *cart* ^*1*^ compared with wild-type flies, whereas phototaxis of both the *e* ^*1*^ and the *ine* ^*MI05077*^ mosaic flies did not differ from that in wild-type flies (Fig 3G). These results suggest the possibility that CarT rather than Ine functions as a carcinine transporter in photoreceptor cells.

### CarT is predominantly localized to photoreceptor terminals

**Fig 4:**
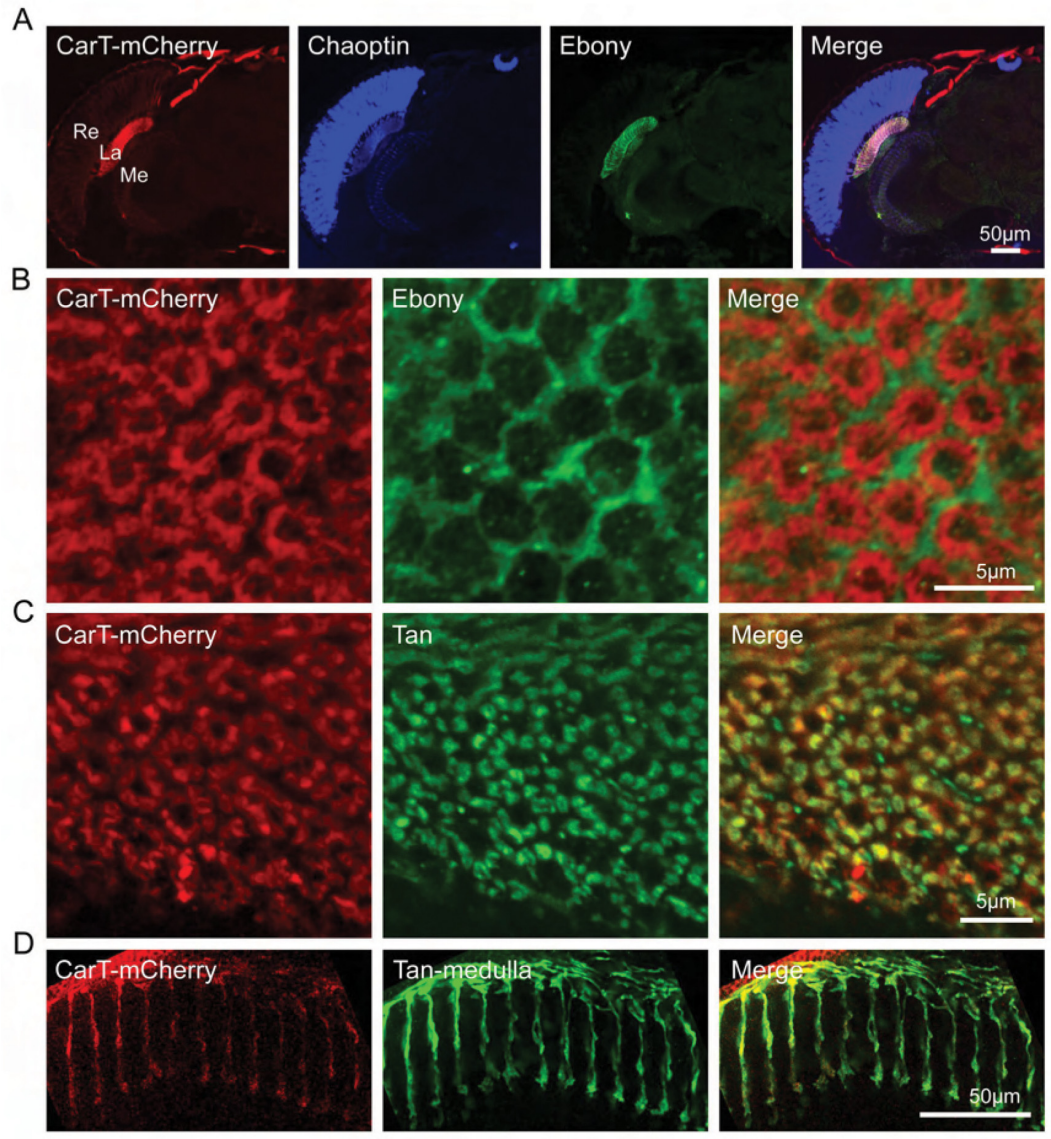
CarT is localized to terminals of photoreceptor neurons. (A) CarT expression was enriched in the lamina and medulla neuropils by a mCherry-tagged transgene labeled with anti-mCherry. *Pcart-cart-mcherry* flies expressing mCherry-tagged CarT driven by the *cart* promoter were used. Cryosections of fly heads were labeled with anti-mCherry (red), anti-Chaoptin (24B10, expressed in photoreceptors) (blue) and anti-Ebony (green, expressed in lamina epithelial glia). La, lamina; Me, medulla; Re, retina. (B) Cross sections of the lamina of *Pcart-cart-mcherry* flies immunolabeled with anti-mCherry (red) and anti-Ebony (green) antibodies showed a complementary pattern. (C) Cross sections of the lamina of *Pcart-cart-mcherry* flies labeled with anti-mCherry (red) and anti-Tan (green) showed an overlapping pattern. (D) Longitudinal sections of the medulla of *Pcart-cart-mcherry* flies labeled with anti-mCherry (red) and anti-Tan (green) showed an overlap in the pattern of R7/R8 labeling.

Trafficking of carcinine into photoreceptors is a key step in histamine recycling in *Drosophila*. As we have proposed here that CarT functions as a carcinine transporter acting at the photoreceptor cell membrane, we examined the localization of CarT to photoreceptor cells to evaluate the cellular location of carcinine transport. Since multiple attempts to generate an anti-CarT antibody failed, we eventually generated transgenic flies that expressed mCherry-tagged CarT driven by the *cart* promoter. Importantly, the *Pcart-cart-mcherry* transgene completely reversed the loss of “on” and “off” transients in *cart* mutant flies (Fig 2C). Although CarT was expressed throughout the photoreceptor neurons, the CarT signal was predominantly detected in the lamina layer where it was marked by the Ebony immunosignal, and not appreciably in the region of the retina (Fig 4A). In cross sections at high magnification we observed that CarT was not co-localized with Ebony to the epithelial glial cells (Fig 4B), but rather co-localized with the photoreceptor cell axon marker Tan to both the lamina and medulla neuropils, to which the R1-R6 and R7/R8 photoreceptors project their axons respectively (Fig 4C and 4D). The finding that CarT expression is enriched in photoreceptor terminals is consistent with the assumption that photoreceptor cells take up carcinine mainly from the local synaptic cleft in the lamina, rather than by a long-distance histamine recycling pathway which is mediated by lamina glia and a retinal pigment cell network [9]. However, we cannot exclude the existence of a long-term trafficking pathway for carcinine.

### Mutant *cart* ^*1*^ shows decreased histamine immunolabeling in photoreceptor terminals

**Fig 5:**
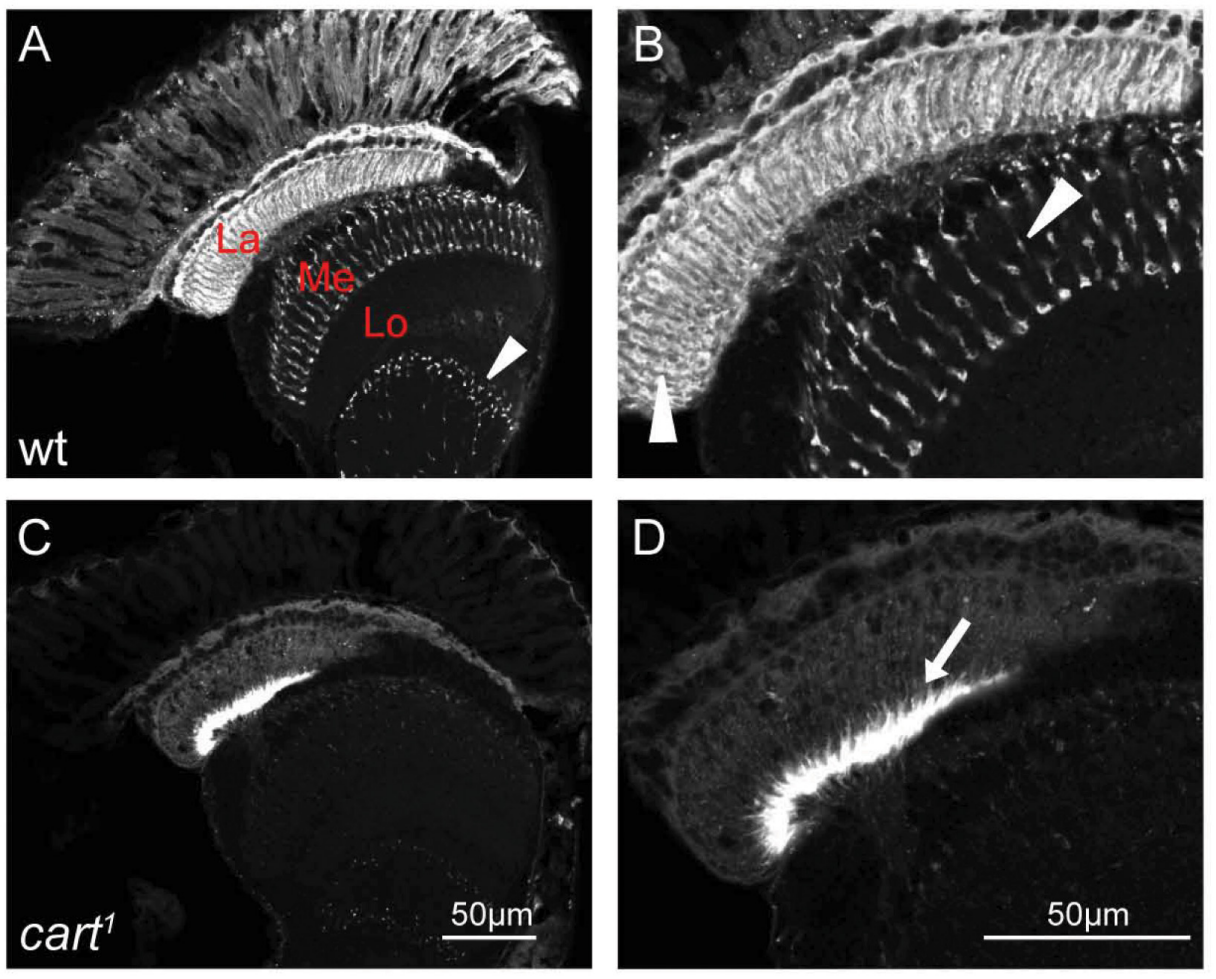
Histamine is reduced in *cart* ^*1*^ mutant photoreceptor neurons. Histamine was immunolabeled in horizontal sections of heads from (A-B) wild type (wt: *w* ^*1118*^) and (C-D) *cart* ^*1*^ mutant flies. (A) Strong signals in the lamina neuropile (La) and R7/R8 terminals in the distal medulla (Me) were detected in control *w* ^*1118*^. Additional immunolabel also appeared from cells in the lobula (Lo) (arrowhead). (B) The enlarged image of the wt head section in (A). Arrowheads in the lamina and medulla identified histamine positive photoreceptor terminals. (C) Loss of photoreceptor histamine signals and strong signals in lamina marginal glia were detected in *cart* ^*1*^ mutant. (D) The enlarged image of the *cart* ^*1*^ head section in (C) showing labeled marginal glia (arrow) but no photoreceptor signals. Scale bars: 50μm

Given that the evidence so far suggests that *cart* acts to transport carcinine into the photoreceptor, where *tan* then acts to hydrolyze it and release histamine, we next sought to examine whether loss of *cart* would decrease histamine labeling. We labeled head cross sections from the *cart* ^*1*^ mutant and from the *w* ^*1118*^ control with anti-histamine antibody. The distribution of histamine signal in *cart* ^*1*^ mutant flies relative to their *w* ^*1118*^ controls reveals a clear loss of photoreceptor signal (Fig 5A and 5B), compatible with the mutant’s inability to take up carcinine and so liberate histamine. In the enlarged images, it is clear that *cart* ^*1*^ mutants showed a dramatic decrease in labeling for histamine in R1-R6 photoreceptor terminals in the lamina, and in R7/R8 photoreceptor terminals in the medulla (Fig 5C and 5D). In contrast to the weak label in R1-R6 photoreceptor terminals in the lamina, a strong label was seen in the underlying marginal glia at the proximal lamina in the *cart* ^*1*^ mutant (Fig 5C and 5D) [3,9,25]. The labeling of this region suggests that histamine might be accumulated at an ectopic site in the *cart* mutant.

### Expression of human OCT2 in photoreceptor cells rescues the defective visual synaptic transmission in *cart* ^*1*^ flies

**Fig 6:**
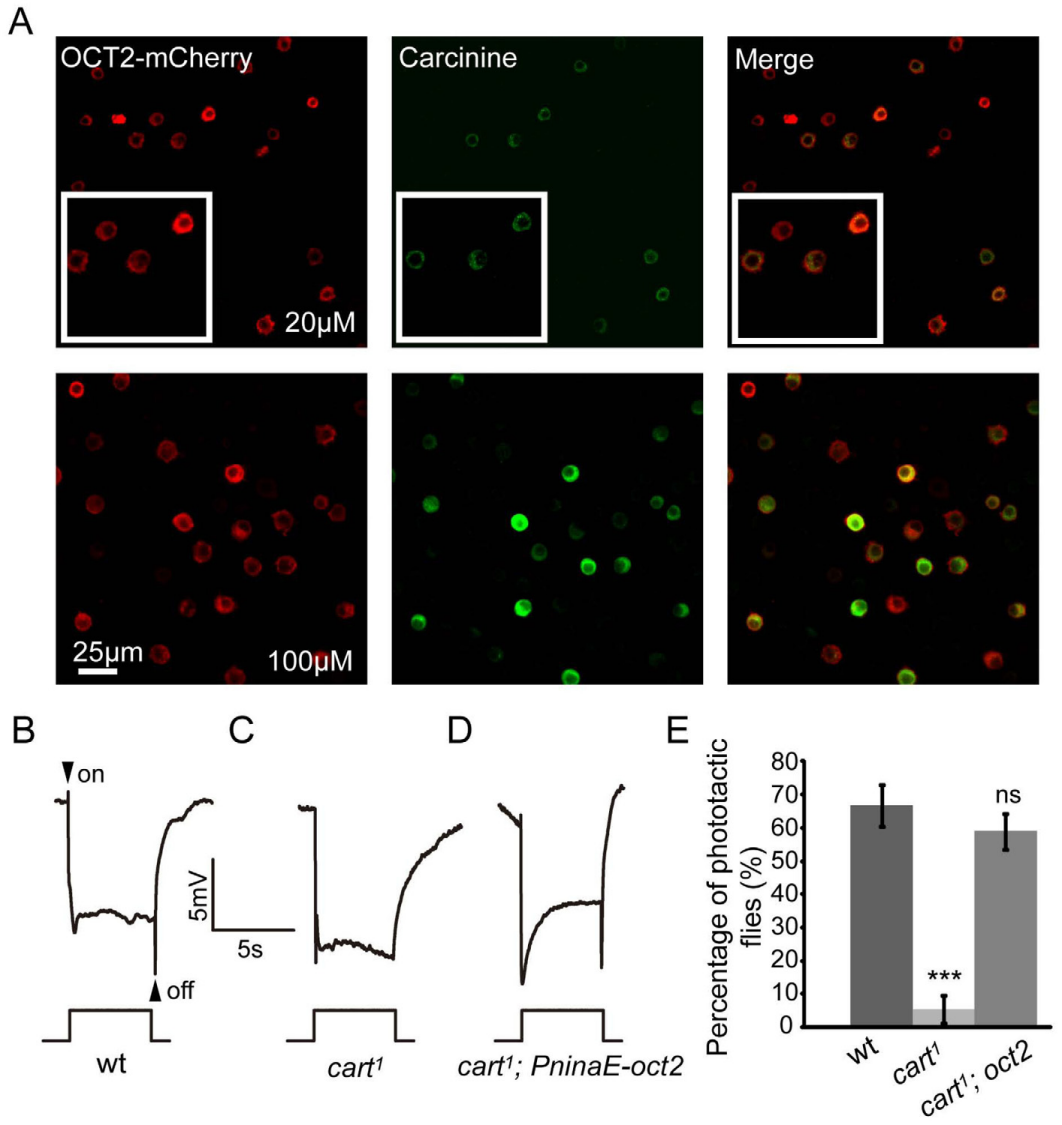
Rescue of *cart* ^*1*^ phenotype by expressing human OCT2 in photoreceptors. (A) OCT2 was able to transport carcinine. S2 cells transiently expressed OCT2-mCherry in a culture medium to which carcinine was added at a final concentration of 20μM or 100 μM. Cells were immunolabeled with anti-carcinine (green), and the mCherry (red) signal was observed directly. The carcinine signal is stronger in the presence of 100μM carcinine. (B-D) ERG recordings from (B) wt: *w* ^*1118*^, (C) *cart* ^*1*^, and (D) *cart* ^*1*^; *PninaE-oct2* flies showed restored “on” and “off” transients after photoreceptor cell-specific expression of human OCT2 in *cart* ^*1*^ flies. (E) Quantification of phototactic behaviors of wt, *cart* ^*1*^, and *cart* ^*1*^; *PninaE-oct2* (*cart* ^*1*^; *oct2*) flies. Error bars represent SD. Significant differences between mutant and wt flies were determined using unpaired *t*-tests (*p < 0.05; **p < 0.01; ***p < 0.001; ns, not significant).

CarT belongs to the SLC22 protein family and is highly homologous to the mammalian OCT2 protein. We therefore wondered whether heterologous expression of OCT2 in *cart* mutant flies would restore the synaptic transmission of photoreceptors. OCT2 is known to mediate low affinity transport of some monoamine neurotransmitters [26]. However, it is not known whether OCT2 is able to transport carcinine. We performed *in vitro* assays to determine whether OCT2 can transport carcinine. After expressing OCT2 in S2 cells, carcinine was taken up by the OCT2-positive cells (Fig 6A). These results indicated that OCT2 can indeed transport carcinine. We next generated a *PninaE-oct2* transgene to express OCT2 in photoreceptor cells only, and introduced this transgene into the *cart* ^*1*^ mutant background. We found that the expression of human OCT2 in *cart* ^*1*^ mutant fly photoreceptor cells fully restored both the “on” and “off” transients and phototaxis in *cart* ^*1*^ flies (Fig 6B–6E). These results demonstrated a conserved function for OCTs in both a mammal and *Drosophila*.

### Histamine metabolite levels are altered in *cart* ^*1*^ mutant flies

**Fig 7:**
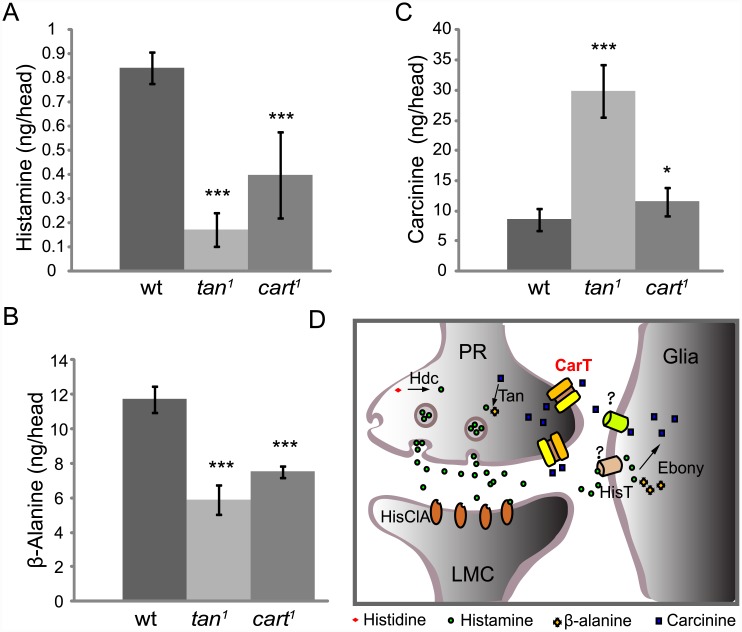
Loss of CarT affects the histamine, β-alanine, and carcinine contents *in vivo*. (A-C) Head histamine, β-alanine, and carcinine contents in the three genotypes indicated. (A-B) The *tan* ^*1*^ and *cart* ^*1*^ mutants had significantly less histamine and β-alanine than wild-type flies (*w* ^*1118*^). (C) The *tan* ^*1*^ mutants had nearly three times as much carcinine as wild-type flies, and *cart* ^*1*^ flies only showed a 35% increase in carcinine content. Error bars indicate SD; significant differences between mutant and wt flies were determined using unpaired t-tests (*p < 0.05; ***p < 0.001). (D) Model of the pathway for histamine recycling. After a light stimulus, the photoreceptor cells (PR) release histamine, synthesized by histidine decarboxylase (Hdc), into the synaptic cleft to activate histamine-gated chloride channels (HisClA) on postsynaptic neurons (LMC). The released histamine is quickly removed by an unknown histamine transporter in epithelial glial cells that express Ebony, and is then deactivated by conjugation to β-alanine. The histamine metabolite carcinine is then transported out of epithelial glial cells (Glia) by a second unknown transporter, and back to photoreceptors by means of the CarT transporter at the photoreceptor cell terminals, where carcinine is then hydrolyzed back into histamine by Tan ready to be pumped into synaptic vesicles in preparation for further release.

We used high-performance liquid chromatography (HPLC) to examine the *in vivo* contents of histamine as well as carcinine and β-alanine, the major metabolites in histamine recycling [18,27]. As expected, in the heads of the *tan* ^*1*^ mutant flies, which are defective in their capacity to hydrolyze carcinine into histamine and β-alanine, the head contents of both histamine and β-alanine were significantly reduced (Fig 7A and 7B). The lack of carcinine uptake by photoreceptor cells in *cart* ^*1*^ mutant flies ultimately depletes carcinine in these cells, which reduces the production of histamine and β-alanine mediated by Tan (Fig 7A and 7B). The reduced head contents of histamine and β-alanine are therefore in agreement with the hypothesis that CarT transports carcinine.

In contrast to histamine and β-alanine, the content of carcinine in the *tan* ^*1*^ mutant heads was approximately three fold higher than the content in wild-type heads, which we interpret to result from diminished hydrolysis of carcinine in photoreceptor cells (Fig 7C). If the flies were not able to transport carcinine into photoreceptor cells for hydrolysis, there should be a greater amount of carcinine in fly heads. As expected, in *cart* ^*1*^ mutants, the head content of carcinine was significantly increased. However, the content of carcinine in the *cart* ^*1*^ mutant was not increased to the same extent as in the *tan* ^*1*^ mutant flies.

## Discussion

Although histamine is an important neurotransmitter known to regulate multiple physiological processes, the mechanism by which histamine content is regulated in the nervous system still remains to be elucidated. Our study identifies a mechanism and pathway for the uptake of a primary metabolite of histamine, which has hitherto defied analysis in any nervous system.

Insofar as histamine is the primary neurotransmitter released by photoreceptors in flies [28], the ease with which photoreceptor function and anatomy can be assayed has made the compound eye the preferred system to study histamine recycling. In particular the eye lends itself readily to the identification of genes that regulate neurotransmission, by enabling comprehensive genetic screens [23,29]. Studies in flies have previously identified a histamine/carcinine recycling pathway that involves two enzymes, Ebony, expressed in the epithelial glia, and Tan, expressed in the photoreceptor cells [13,30]. However, the key neurotransmitter transporters required for the histamine/carcinine shuttle pathway have not been identified. Conceptually, the putative carcinine transporter should be functionally coupled to Tan for the uptake of carcinine into photoreceptor cells and its subsequent hydrolysis. For this, both should colocalize to photoreceptors as we have shown in this study.

In this study, we identified a new SLC22A family protein CarT and provided evidence that it is functionally coupled with Tan as a photoreceptor cell-enriched carcinine transporter. CarT is predominantly localized to photoreceptor terminals and is able to transport carcinine *in vitro*. The decrease in head histamine and β-alanine and the increase in head carcinine in *cart* ^*1*^ support this hypothesis. The reduction in histamine content in *cart* ^*1*^ mutants is ~60%. This amount corresponds rather closely to the reduction in head histamine seen in the mutant *sine oculis*, which lacks compound eyes and has 28% of the histamine found in the wild-type [27]. The reduction in *sine oculis* suggests that residual head histamine is not located in the compound eye visual system. In the same way, the reduction in *cart* ^*1*^ is not accessible to photoreceptor synaptic transmission. Moreover, mutant *ebony*, in which head histamine content is reduced by 50%, has an abnormal ERG and phototaxis, corresponding to the strong ERG and phototaxis defects seen in the *cart* ^*1*^ mutant. Consistent with these HPLC data, we also found a clear difference in the immunosignal for histamine between *cart* ^*1*^ and control fly photoreceptors. The *cart* head increase in carcinine is not as high as that observed in the head of *tan* mutants, which raises the question of why the increases in carcinine in *tan* and *cart* mutant flies are not similar. This may be because carcinine released in the synaptic cleft in *cart* ^*1*^ mutants is removed by other cells, or alternatively it may enter the hemolymph and be excreted. Consistent with the latter, the carcinine content in the abdomen is increased by 43% in *cart* ^*1*^ compared with control *w* ^*1118*^ flies. We cannot address the carcinine transport by other cells in the lamina, in particular the epithelial and marginal glia, which surround the photoreceptor terminals and which contain carcinine [18], which we propose must therefore express other carcinine transporters.

All of the known plasma membrane neurotransmitter transporters are members of the Solute Carrier (SLC) family of proteins [31]. The most extensively studied of these transporters are members of the SLC6 subfamily, a group of Na^+^/Cl^-^—dependent transporters for serotonin, dopamine, norepinephrine, GABA and glycine [32]. OCTs, which belong to the SLC22 subfamily, are known to mediate sodium-independent transport of positively charged organic compounds [33]. The expression of OCT2 in neurons has been evaluated previously, but the neuronal function of OCT2 has not been explored sufficiently [26,33,34]. Carcinine has been identified as a native metabolite related to histamine in multiple tissues in mammals, where it may serve as an antioxidant for scavenging toxic active oxygen species, especially in retinal photoreceptors [35,36]. Our findings that OCT2 can transport the inactive histamine metabolite carcinine both *in vitro* and *in vivo* suggests a possible new mechanism for OCTs to function in neurotransmitter recycling and cell protection.

The histamine/carcinine shuttle pathway plays a dominant role in maintaining an adequate level of histamine in photoreceptors. Evidence for the direct uptake of histamine into photoreceptor cells is lacking, insofar as Ebony is necessary to rescue ERG transients in histamine-fed *hdc* mutant flies [37]. In addition, in our model S2 cells expressing CarT fails to transport histamine, providing further support for the hypothesis that direct uptake of histamine into the photoreceptor terminals may not occur. Although the enzymatic deconjugation of carcinine to yield histamine has been well established, the route through which carcinine is then trafficked back to the photoreceptor has not been established. It has been suggested that recycling of carcinine to photoreceptor cells involves a long-distance pathway mediated by a gap-junction dependent network of lamina and retinal pigment cells [9]. In our study, we observed that CarT is predominantly localized to the terminals of photoreceptor neurons, rather than to their cell bodies in the retina layer, which suggests that carcinine is transported back to photoreceptor cells mainly from the synaptic cleft in the lamina (Fig 7D). It is also possible that this local pathway works in parallel with the long-distance neurotransmitter recycling pathway. Finally, data from the current study together with previous reports now provide evidence for a more complete histamine/carcinine recycling pathway, one which is critical for maintaining the normal histamine content of neurons (Fig 7D).

To complete the model of how histamine is recycled in the fly’s eye (Fig 7D), the remaining question concerns how histamine is transported into the epithelial glia and how carcinine is then transported out of the glia. No specific histamine transporter has been found, in either insects or vertebrates. In insects a mechanism for the fast removal of histamine from the synaptic cleft is essential to maintain the rapid signaling required for insect vision. One transporter may be White [38], but the problem is that in eukaryotes all known ABC transporters move substrates in the opposite direction i.e. out of the cell. To complete the return path for the carcinine will require us to identify how carcinine is exported out of the epithelial glia. To identify the transporter for this function it will be necessary to identify genes, for example from the expression of mRNA transcripts of genes, such as ebony [12, 13], that are enriched in the epithelial glia, in an approach that parallels the one we have adopted here to identify CarT. The transport of β-alanine, the other substrate needed for carcinine synthesis, seems to be of minor importance because this amino acid is present in the head in concentrations greatly exceeding those needed for histamine recycling and it can be also easily synthesized on demand from aspartate or uracil. Answering these questions is necessary to complete the current scheme (Fig 7D) for the recycling of histamine, to which our findings now identify CarT as the photoreceptor uptake transporter.

## Methods

### Fly stocks

The following stocks were obtained from the Bloomington Stock Center: (1) 122, *e* ^*1*^; (2) 130, *tan* ^*1*^; (3) 38094, *ine* ^*MI05077*^; (4) 3605, *w* ^*1118*^; and (5) 24749, *M(vas-int*.*Dm)ZH-2A;M(3xP3-RFP*.*attP)ZH-86Fb*. The *(nos-Cas9)attP2* flies were obtained from the lab of Dr. J. Ni at Tsinghua University, Beijing, China. The *ey-flp;GMR-hid CL FRT40A/Cyo*, *ey-flp;FRT42D GMR-hid CL/Cyo*, *GMR-hid CL FRT19A/FM7;ey-flp*, and *ey-flp;FRT82B GMR-hid CL /TM3* flies were maintained in the lab of Dr. T. Wang at the National Institute of Biological Sciences, Beijing, China.

### Generation of plasmid constructs and transgenic flies

The *cart*, *CG3790*, *ine*, and *Hdc* cDNA sequences were amplified from GH05908, GH20501, LP16156, and LD44381 cDNA clones obtained from DGRC (Drosophila Genomics Resource Center, Bloomington, IN, USA). The *oct2* cDNA sequences were amplified from IOH56335 cDNA clones obtained from Ultimate^™^ ORF clones (Thermo Fisher Scientific, Waltham, USA). Their entire CDS sequences, excluding the stop codon, were subcloned into the *pIB-cmcherry* vector (Invitrogen, Carlsbad, USA) for expression in S2 cells. To construct *PninaE-cart*, *PrdhB-cart*, and *PninaE-oct2*, the entire coding region of *cart* and *oct2* was amplified from cDNA clones and cloned into the *pninaE-attB* and *prdhB* vectors (both gifts from the lab of Dr. C. Montell at the University of California, Santa Barbara, USA)[17,24,39]. To construct *Pcart-cart-mcherry*, the promoter region (-2579 to +11 base pairs 5' to the transcription start site) of the *cart* gene was amplified from genomic DNA, and *cart-mcherry* was amplified from *pIB-cart-mcherry*. These constructs were injected into *M(vas-int*.*Dm)ZH-2A;M(3xP3-RFP*.*attP)ZH-86Fb* embryos, and transformants were identified on the basis of eye color. The *(3xP3-RFP*.*attP)* locus was removed by crossing with *P(Crey)* flies.

### Generation of the *cart* mutant flies

The *cart* ^*1*^ and *cart* ^*2*^ mutations were generated using the Cas9/sgRNA system as described previously [19]. Three recognition sequences of guiding RNA to the *cart* locus were designed with tools available at the following website http://www.flyrnai.org/crispr2/ (sgRNA1: AAAACCGCACGGTATGCAGG, sgRNA2: CCTGTCCGGCGTCACTTATC, sgRNA3: TGAGCGTCATGGACACCCAG). These were cloned into the *U6b-sgRNA-short* vector. The *pU6-sgRNA1* and *pU6-sgRNA2* plasmids were used to generate the *cart* ^*1*^ mutant flies, while *pU6-sgRNA1* and *pU6-sgRNA3* were used to generate the *cart* ^*2*^ mutant flies. Plasmids were injected into the embryos of *(nos-Cas9)attP2* flies. The F1 progeny were screened by PCR to identify the *cart* ^*1*^ and *cart* ^*2*^ deletions, using the following primers:

pF: 5’-TGTCGCTACAAATCTTAGATCCAA-3'

pR: 5’-CCATGTCAGATATTGAGGACAACG-3’

### Electroretinogram recordings

Two glass microelectrodes filled with Ringer’s solution were inserted into small drops of electrode cream (Sigma, New Jersey, USA) placed on the surfaces of the compound eye and the thorax. A Newport light projector (model 765) was used for stimulation. The source light intensity was 2000lux, and the light color was orange (the source light was filtered by FSR-OG550 filter). ERG signals were amplified with a Warner electrometer IE-210 and recorded with a MacLab/4 s A/D converter and the clampelx 10.2 program (Warner Instruments, Hamden, USA). All recordings were carried out at 23°C.

### Carcinine/histamine transport assay

S2 cells were grown in Schneider’s *Drosophila* medium with 10% Fetal Bovine Serum (Gibco, Carlsbad,USA), and transfected with vigofect reagent (Vigorous Biotechnology, Beijing, China). Carcinine or histamine was added to the medium to yield final concentrations as indicated in the Figure legends. After incubation for 3h, S2 cells were transferred to poly-L-lysine-coated slices, fixed with 4% paraformaldehyde(for carcinine immunolabeling) or 4% 1-ethyl-3-(3-dimethylaminopropyl) carbodiimide (EDAC)(for histamine immunolabeling) for 30min at 25°C, and incubated with rabbit anti-carcinine/histamine (1:100, ImmunoStar, USA)[9] or rat anti-carcinine antibodies (1:100, raised by Dr. Gabrielle Boulianne, from the lab of Dr. I. A Meinertzhagen) [18]. Goat anti-rabbit lgG conjugated to Alexa 488 (1:500, Invitrogen, CA) and goat anti-rat lgG conjugated to Alexa 488 (1:500, Invitrogen, CA) were used as secondary antibodies, and images were recorded with a Nikon A1-R confocal microscope.

### Immunohistochemistry

Fly heads were fixed with 4% paraformaldehyde for 2h at 4°C or 4% EDAC (for histamine staining), and immersed in 12% glucose overnight at 4°C. The heads were embedded in O.C.T^™^ compound (Tissue-Tek, Torrance, USA), and 10μm thick cryosections were cut. Immunolabeling was performed on cryosections sections with mouse anti-24B10 (1:100, DSHB, http://dshb.biology.uiowa.edu/), rat anti-RFP (1:200, Chromotek, Martinsried, Germany), rabbit anti-Ebony (1:200, lab of Dr. S. Carroll, University of Wisconsin, Madison, USA), and anti-Tan (1:200, lab of Dr. B. Hovemann, Ruhr Universität Bochum, Germany) [30] as primary antibodies. For histamine staining, rabbit anti- histamine (1:100, ImmunoStar, USA) was used as a primary antibody. The antibody was preadsorbed with carcinine as previously reported [9]. Goat anti-rabbit lgG conjugated to Alexa 488 (1:500, Invitrogen, USA), goat anti-rat lgG conjugated to Alexa 568 (1:500, Invitrogen, USA) and goat anti-mouse lgG conjugated to Alexa 647 (1:500, Jackson ImmunoResearch, USA) were used as secondary antibodies. The images were recorded with a Nikon A1-R confocal microscope.

### The phototaxis assay

A transparent glass tube of 20 cm long and 2.5 cm in diameter was used in this assay. A white light source (with a light intensity of 6000lux) was put at one end of the glass tube, and dark-adapted flies were collected and gently tapped into the other end of the tube. The tube was placed horizontally in the dark, and we counted the number of flies that walked past an 11-cm mark on the tube within 90s after turning the light on. Phototaxis was calculated by dividing the number of flies that walked past the mark as a proportion of the total number of flies. These assays were performed under dark conditions. To quantify the phototactic behaviors of each genotype, three groups of flies were collected for each genotype and three repeats made for each group. Each group contained ≥ 20 flies. Results were expressed as the mean of the mean values for the three groups.

### RNA extraction and qPCR

Total RNA was prepared from the heads of three-day-old flies using Trizol reagent (Invitrogen, Carlsbad, USA), followed by TURBO DNA-free DNase treatment (Ambion, Austin, USA). Total cDNA was synthesized using an iScript cDNA synthesis kit (Bio-Rad Laboratories, USA). iQ SYBR green supermix was used for the real-time PCR (Bio-Rad Laboratories, USA). Three different samples were collected from each genotype. The primers used for qPCR were as follows:

*ninaE-fwd*, 5’-ACCTGACCTCGTGCGGTATTG-3’

*ninaE-rev*, 5’-GGAGCGGAGGGACTTGACATT-3’

g*pdh-fwd*, 5’-GCGTCACCTGAAGATCCCATG-3’

*gpdh-rev*, 5’-CTTGCCATACTTCTTGTCCGT-3’

*rdhB-fwd*, 5’-TTGAGGCACTCAGGGATCAAG-3’

*rdhB-rev*, 5’-CACCACATTCGTGTCGAACAG-3’

*cart-fwd*, 5’-TACAGCACAAGGGTCTCATCC-3’

*cart-rev*, 5’-AGACCATCCTAATCACGCTGAG-3’

### High-performance liquid chromatography (HPLC)

To measurement the total head contents of histamine, β-alanine, and carcinine, flies were decapitated and their heads collected as previously reported [10]. The heads were then processed and analyzed using HPLC with electrochemical detection, all as previously reported [18,27]. Each sample contained ~50 *Drosophila* heads, and the mean values from five samples were calculated.

## Supporting Information

S1 Fig CG9317 is an SLC22 family protein. Alignment of the *Drosophila* CG9317 amino acid sequence with *Drosophila* CG3790, mouse OCT2, and mouse OCT3. Identical residues, found in at least two proteins, are enclosed in black boxes. CG9317 is 32% identical to OCT2 and 31% identical to OCT3, whereas CG3790 is 30% identical to OCT2 and 29% identical to OCT3. The transmembrane domains are indicated by solid lines above the corresponding sequences. The running tally of amino acids is indicated to the right.
(EPS)

S2 Fig CG9317, CG3790, and Ine cannot transport histamine. S2 cells transiently expressed (A) mCherry, (B) Ine-mCherry, (C) CG3790-mCherry, (D) CG9317-mCherry or (E) mCherry and Hdc. Histamine was added to the culture medium at a final concentration of 20μM. Cells labeled with anti-histamine (green) and DAPI (blue), or mCherry (red) were observed directly. Scale bar, 25μm
(EPS)

S3 Fig Confirming the carcinine transporter activity of CG9317 in S2 cells. S2 cells transiently expressed (A) mCherry, (B) Ine-mCherry, (C) CG3790-mCherry, or (D) CG9317-mCherry. Carcinine was added to the S2 cells culture medium at a final concentration of 50μM. Cells labeled with rat anti-carcinine (green) and DAPI (blue), and the mCherry (red) signal was observed directly. Scale bar, 20μm.
(EPS)

S4 Fig The verification of *cart* ^*1*^ and *cart* ^*2*^ loci by DNA sequencing. DNA sequencing results of the Cas9-mediated break points in (A) *cart* ^*1*^ and (B) *cart* ^*2*^ flies. The representative DNA sequence of the wild-type locus (lower trace) shows the break points of the deletions generated. Upstream sequences of the 5’ breakpoints are marked in black, and downstream sequences of 3’ breakpoint are marked in red. The genomic positions corresponding to the break points are indicated.
(EPS)
